# A bibliometric analysis of sepsis-induced myocardial dysfunction from 2002 to 2022

**DOI:** 10.3389/fcvm.2023.1076093

**Published:** 2023-01-30

**Authors:** Hanyi Yao, Shufang Liu, Zhiyu Zhang, Zixi Xiao, Dongping Li, Zhangqing Yi, Yuyang Huang, Haojie Zhou, Yifeng Yang, Weizhi Zhang

**Affiliations:** ^1^Department of Cardiovascular Surgery, The Second Xiangya Hospital, Central South University, Changsha, China; ^2^Clinical Center for Gene Diagnosis and Therapy, The Second Xiangya Hospital, Central South University, Changsha, China; ^3^Department of Cardiovascular Medicine, The Second Xiangya Hospital, Central South University, Changsha, China

**Keywords:** sepsis-induced myocardial dysfunction, bibliometric, CiteSpace, VOSviewer, visual analysis

## Abstract

**Background:**

Sepsis-induced myocardial dysfunction (SIMD) has a significant contribution to sepsis-caused death in critically ill patients. In recent years, the number of published articles related to SIMD has increased rapidly. However, there was no literature that systematically analyzed and evaluated these documents. Thus, we aimed to lay a foundation for researchers to quickly understand the research hotspots, evolution processes and development trends in the SIMD field *via* a bibliometric analysis.

**Methods:**

Articles related to SIMD were retrieved and extracted from the Web of Science Core Collection on July 19th, 2022. CiteSpace (version 6.1.R2) and VOSviewer (version 1.6.18) were used for performing visual analysis.

**Results:**

A total of 1,076 articles were included. The number of SIMD-related articles published each year has increased significantly. These publications mainly came from 56 countries, led by China and the USA, and 461 institutions, but without stable and close cooperation. As authors, Li Chuanfu published the most articles, while Rudiger Alain had the most co-citations. Shock was the journal with the most studies, and Critical Care Medicine was the most commonly cited journal. All keywords were grouped into six clusters, some of which represented the current and developing research directions of SIMD as the molecular mechanisms.

**Conclusion:**

Research on SIMD is flourishing. It is necessary to strengthen cooperation and exchanges between countries and institutions. The molecular mechanisms of SIMD, especially oxidative stress and regulated cell death, will be critical subjects in the future.

## 1. Introduction

According to the latest recommendations, called sepsis-3, sepsis is defined as a life-threatening organ dysfunction caused by a dysregulated host response to infection (1). Sepsis is one of the most important causes of death in critically ill patients, and sepsis-induced myocardial dysfunction (SIMD) has a significant contribution to this situation (2). The cardiac function of patients with SIMD can return to a normal level gradually after the primary disease is cured, so SIMD is considered as a functional cardiac disease secondary to sepsis (3). According to evidence, more than forty percent of patients with severe sepsis and septic shock have cardiac involvement (4), and myocardial dysfunction increases mortality in such patients by about fifty percent (5). In addition, SIMD is also an independent predictor of prognosis for patients (6).

In recent years, the number of published articles related to SIMD has increased rapidly. However, there is no literature that systematically analyzed and evaluated these documents. Bibliometrics, first proposed by Alan Pritchard in 1969, can analyze a large number of documents in a designated field of study by using mathematical and statistical methods (7). As a result, it can assist researchers in quickly understanding the research status and hotspots in the field, as well as forecasting its development trend (8). Based on this, many researchers have used bibliometric analysis to study their respective domains (9–12).

In this study, we used CiteSpace (version 6.1.R2) and VOSviewer (version 1.6.18) to summarize the hotspots and reveal the development trends of SIMD in the past 20 years. It aimed to lay a foundation for researchers to quickly understand the research hotspots, evolution processes and development trends in the SIMD field.

## 2. Materials and methods

### 2.1. Data collection

**FIGURE 1:**
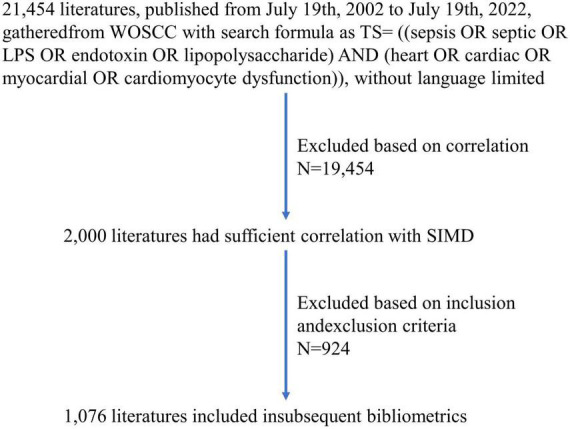
Flowchart of literature-allocation.

Data was retrieved and extracted from the Web of Science Core Collection (WOSCC) on July 19th, 2022. TS = [(sepsis OR septic OR LPS OR endotoxin OR lipopolysaccharide) AND (heart OR cardiac OR myocardial OR cardiomyocyte dysfunction)] was entered into the search formula. The retrieval time range was from July 19th, 2002 to July 19th, 2022, and language was not limited. A total of 21,454 papers (no duplicates) were gathered, including 19.055 articles and 2,399 reviews. In order to ensure sufficient correlation between literature content and SIMD and reduce the number of literatures to be screened, we used correlation ranking in WOSCC and selected the top 2,000 literatures for detailed screening. These papers were screened according to the following criteria by two independent researchers. Inclusion criteria: (1) the title of the literature deals with SIMD or its synonyms; (2) the background of the literature is related to SIMD; (3) the subjects were patients with SIMD, or the materials were animal or cell models of SIMD; (4) the purpose and conclusion of the literature are related to the mechanism, etiology, diagnosis, treatment or prognosis of SIMD. Exclusion criteria: (1) the main content and central idea of the literature have little association with SIMD; (2) the type of the literature is review, case report or meta-analysis; (3) the content of the literature is clinical treatment guidelines or consensus; (4) WOSCC could not provide complete information about the literature, including title, abstract, keywords, authors and institutions. Articles only need to meet one of these requirements to be included or excluded. Finally, after checking, a total of 1,076 eligible articles were decided for subsequent analysis and were exported in the form of “Full Record and References” and saved as a plain text file named download_txt (Figure 1).

### 2.2. Data analysis

All relevant data were gathered from the WOSCC. Microsoft Office Excel 2019 was used to manage data, analyze and visualize the annual distributions of articles. CiteSpace (version 6.1.R2) and VOSviewer (version 1.6.18) were used for performing visual analysis, including the distribution of institutions, contributions of authors, core journals and viewers of keywords and timelines.

CiteSpace is a JAVA-based computer system developed by Chaomei Chen for data analysis and visualization (13). Since its publication, CiteSpace has become one of the most commonly used visual analysis software in bibliometrics (11). It provides multiple perspective analyses for the particular study field to present the rules and distribution in visualization, which help researchers visually distinguish the research hotspots and the development process of the field and predict future development trends (14).

VOSviewer is a software for graphical representation of bibliometric maps developed by Nees Jan van Eck and Ludo Waltman (15). Compared with SPSS, this program is more suitable for large maps because of its powerful graphics processing capabilities. Moreover, its ability to density map is irreplaceable in current bibliometrics (16).

## 3. Results

### 3.1. The annual growth trend of publications

**FIGURE 2:**
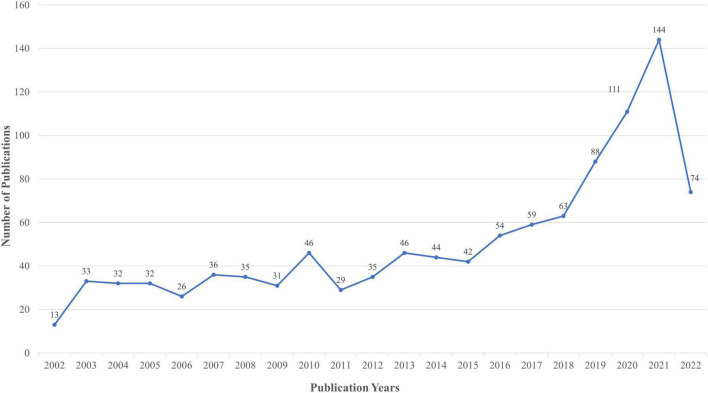
Trend of the number of literatures of SIMD in the past 20 years.

The number of papers concerning SIMD was counted on an annual basis, which showed an increasing trend (Figure 2). In 2002, there were fewer articles related to SIMD. From 2003 to 2015, the popularity of SIMD was relatively stable, the number of related articles fluctuated at a low level, and the overall increase was very slight. From 2016 to 2018, the annual number of related articles published increased, which indicates that more researchers paid attention to SIMD in that period. Since 2018, the number of SIMD-related research results has increased rapidly, which trend is evident, although data for 2022 is not fully collected because of the deadline for data collection in our study.

### 3.2. Distribution of countries/regions and institutions

**FIGURE 3:**
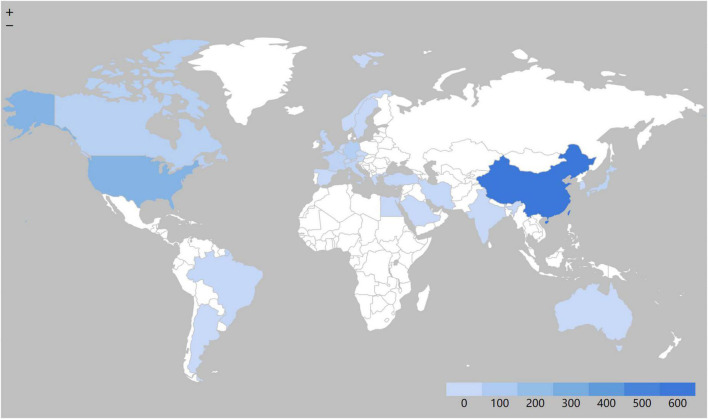
The distribution map of countries/regions.

A total of 1,076 articles were co-authored by researchers from 56 countries and 461 institutions. The country with the largest number of publications is China (551, 51.21%), with the USA (253, 23.51%) in second place, followed by Germany (77, 7.16%), Canada (64, 5.95%), and other countries (Table 1 and Figure 3). Among the top 10 countries, the USA (0.38), Germany (0.37), and France (0.3) have higher centrality, while China (0.17), Italy (0.15), and England (0.1) are in the second tier (Table 1), which is inconsistent with the ranking of the number of publications. The institution that published the most articles was Wuhan Univ (37, 3.44%), followed by China Med Univ (25, 2.32%), Nanjing Med Univ (23, 2.14%), Jinan Univ (22, 2.04%), and others (Table 1). Notably, the top 10 institutions are all from China.

**TABLE 1:** The top 10 countries/regions and institutions related to SIMD.

Rank	Country/Region	Year	Centrality	Count (%)	Institution	Year	Centrality	Count (%)
1	PEOPLES R CHINA	2003	0.17	551 (51.21%)	Wuhan Univ	2014	0.02	37 (3.44%)
2	USA	2002	0.38	253 (23.51%)	China Med Univ	2008	0.12	25 (2.32%)
3	GERMANY	2002	0.37	77 (7.16%)	Nanjing Med Univ	2009	0.12	23 (2.14%)
4	CANADA	2002	0.08	64 (5.95%)	Jinan Univ	2006	0.06	22 (2.04%)
5	JAPAN	2002	0.06	44 (4.09%)	Fourth Mil Med Univ	2009	0.05	20 (1.86%)
6	FRANCE	2003	0.3	42 (3.90%)	Harbin Med Univ	2013	0.05	15 (1.39%)
7	ITALY	2003	0.15	35 (3.25%)	Huazhong Univ Sci and Technol	2007	0.01	15 (1.39%)
8	ENGLAND	2002	0.1	33 (3.07%)	Southern Med Univ	2019	0.07	14 (1.30%)
9	BRAZIL	2003	0	18 (1.67%)	Cent South Univ	2010	0.04	14 (1.30%)
10	TURKEY	2004	0.01	15 (1.39%)	Fudan Univ	2007	0.09	14 (1.30%)

**FIGURE 4:**
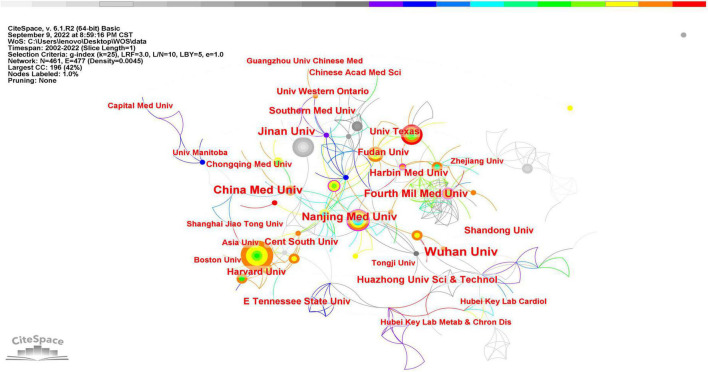
CiteSpace visualization map of institutions related to SIMD.

In Figure 4, institutions are represented by nodes, and the cooperative relationships between institutions are represented by the lines between nodes. The color and thickness of the lines represent the time and degree of cooperation, respectively. It is clear that cooperation between institutions has been fairly evenly distributed over the past 20 years. However, there is a lack of stable and close cooperation between the vast majority of institutions.

### 3.3. Authors and co-cited authors

**FIGURE 5:**
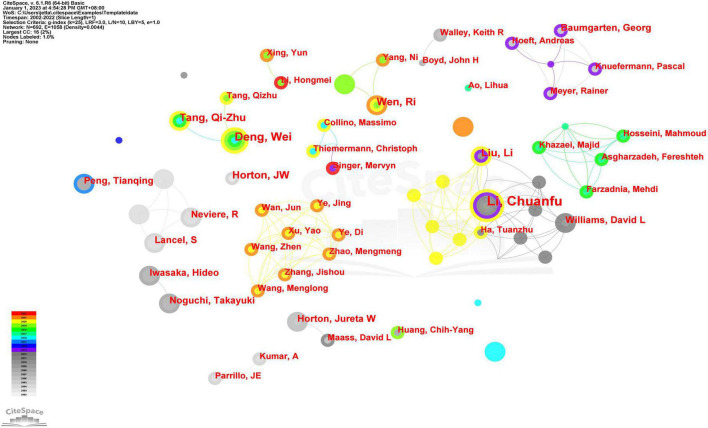
CiteSpace visualization map of authors related to SIMD.

A total of 694 authors contributed to the publication of the 1,076 articles included in this analysis. Li Chuanfu (12, 1.12%) had published the most, followed by Deng Wei (8, 0.74%), Tang Qizhu (7, 0.65%), and others (Table 2). However, all of the top 10 authors had low centrality (0). It seemed to suggest that the contributions and influence of authors in the field of SIMD were scattered, and that there was no author acting as a bridge character. Unlike institutions, authors formed many independent collaborating teams with close cooperation, such as the team centered on Li Chuanfu and Liu Li, on Deng Wei and Tang Qizhu, and on Neviere R and Lancel S (Figure 5). In addition, it was interesting and reasonable that the same author might belong to different teams in different years, like Wen Ri for example.

**TABLE 2:** The top 10 authors and co-cited authors related to SIMD.

Rank	Authors	Centrality	Count (%)	Co-cited author	Centrality	Citation
1	LI CHUANFU	0	12 (1.12%)	Rudiger Alain	0.01	238
2	DENG WEI	0	8 (0.74%)	Kumar Anand	0.05	224
3	TANG QI-ZHU	0	7 (0.65%)	Singer Mervyn	0.02	224
4	WEN RI	0	6 (0.56%)	Parrillo Joseph E	0.04	212
5	LIU LI	0	6 (0.56%)	Merx Marc W	0.04	195
6	HORTON JW	0	6 (0.56%)	Parker Margaret M	0.08	188
7	NEVIERE R	0	5 (0.46%)	Angus Derek C	0.04	157
8	BAUMGARTEN GEORG	0	5 (0.46%)	Hotchkiss Richard S	0.04	147
9	IWASAKA HIDEO	0	5 (0.46%)	Vieillard-baron A	0.03	121
10	LANCEL S	0	5 (0.46%)	Zanotti-cavazzoni SL	0.02	112

Co-cited authors are different authors cited in one or more articles simultaneously. Only seven of the 1,058 cited authors from 1,076 analyzed objects were cited more than 150 times. The most frequently co-cited authors were Rudiger Alain (238), Kumar Anand (224), Singer Mervyn (224), Parrillo Joseph E (212), and etc. (Table 2).

### 3.4. Journals and co-cited journals

VOSviewer (version 1.6.18) was used to perform visual analyses of published journals and co-cited journals.

The 1,076 papers included in the analysis were published in 361 journals. The journal *Shock* (68, 6.32%) occupied the largest number (Table 3). Only *Shock*, *Critical Care Medicine* (44, 4.09%) and *American Journal of Physiology-Heart and Circulatory Physiology* (32, 2.97%) published more than 30 articles. Besides, according to the 2021 journal citation reports (JCR), the top 10 journals were almost evenly distributed among Q1 (3, 30%), Q2 (4, 40%), and Q3 (3, 30%). *Cardiovascular Research* (13.081) had the highest impact factor (IF) of these journals.

**TABLE 3:** Top 10 journals and co-cited journals related to SIMD.

Rank	Journal	Count (%)	IF (2021)	JCR (2021)	Co-cited journal	Citation	IF (2021)	JCR (2021)
1	Shock	68 (6.32%)	3.533	Q3	Critical Care Medicine	719	9.296	Q1
2	Critical Care Medicine	44 (4.09%)	9.296	Q1	Circulation	620	39.918	Q1
3	American Journal of Physiology-heart and Circulatory Physiology	32 (2.97%)	5.125	Q2	Shock	573	3.533	Q3
4	Molecular Medicine Reports	24 (2.23%)	3.423	Q3	American Journal of Physiology-heart and Circulatory Physiology	553	5.125	Q2
5	Plos One	23 (2.14%)	3.752	Q2	Journal of Molecular and Cellular Cardiology	502	5.763	Q2
6	Journal of Molecular and Cellular Cardiology	21 (1.95%)	5.763	Q2	Circulation Research	495	23.213	Q1
7	Inflammation	20 (1.86%)	4.657	Q3	Journal of Biological Chemistry	440	5.486	Q2
8	International Immunopharmacology	17 (1.58%)	5.714	Q2	Cardiovascular Research	415	13.081	Q1
9	Cardiovascular Research	16 (1.49%)	13.081	Q1	Journal of Clinical Investigation	397	19.456	Q1
10	Frontiers in Pharmacology	16 (1.49%)	5.988	Q1	Critical Care	362	19.334	Q1

To some extent, the number of citations that a journal receives indicates its influence in a particular field. In the last 20 years, eight journals have been cited more than 400 times (Table 3). The most co-cited journal was *Critical Care Medicine* (719), followed by *Circulation* (620), *Shock* (573), *American Journal of Physiology-Heart and Circulatory Physiology* (553), *Journal of Molecular and Cellular Cardiology* (502), and etc. Meanwhile, sixty percent of the top 10 co-cited journals were in Q1 region.

**FIGURE 6:**
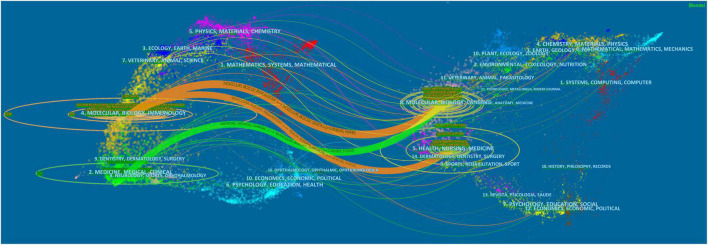
The dual-map overlay of journals on SIMD.

The dual-map overlay of journals can clearly show the distribution of journals and the citation relationship between journals on the left and corresponding co-cited journals on the right (Figure 6). In this way, three main reference paths were identified. It indicated that papers published in Molecular/Biology/Genetics journals were often cited by Molecular/Biology/Immunology and Medicine/Medical/Clinical journals, and that papers published in Health/Nursing/Medicine were also often cited by Molecular/Biology/Immunology.

### 3.5. Co-cited references and references burst

CiteSpace was used to analyze the co-cited references. As shown in Table 4, *The Third International Consensus Definitions for Sepsis and Septic Shock (Sepsis-3)* (110) had a much higher number of citations than other cited references. *Mechanisms of Sepsis-Induced Cardiac Dysfunction* (0.36) had the best centrality, and the centrality of *Cardiac Dysfunction in Severe Sepsis and Septic Shock* (0.11) was also a little greater than 0.10.

**TABLE 4:** Top 10 co-cited references related to SIMD.

Rank	Title	Citation	Year	Centrality	References
1	The Third International Consensus Definitions for Sepsis and Septic Shock (Sepsis-3)	110	2016	0.01	(1)
2	Sepsis-Induced Myocardial Dysfunction: Pathophysiology and Management	50	2016	0.01	(29)
3	Sepsis-Induced Cardiomyopathy: Mechanisms and Treatments	49	2017	0.06	(30)
4	Mechanisms of Sepsis-Induced Cardiac Dysfunction	37	2007	0.36	(32)
5	Septic Cardiomyopathy	37	2018	0.01	(33)
6	Beclin-1-Dependent Autophagy Protects the Heart during Sepsis	33	2018	0.01	(19)
7	Sepsis and the Heart	31	2007	0.03	(6)
8	Cardiac Dysfunction in Severe Sepsis and Septic Shock	30	2009	0.11	(34)
9	STING-IRF3 Contributes to Lipopolysaccharide-Induced Cardiac Dysfunction, Inflammation, Apoptosis and Pyroptosis by Activating NLRP3	30	2019	0.01	(35)
10	The Septic Heart Current Understanding of Molecular Mechanisms and Clinical Implications	30	2019	0	(37)

**FIGURE 7:**
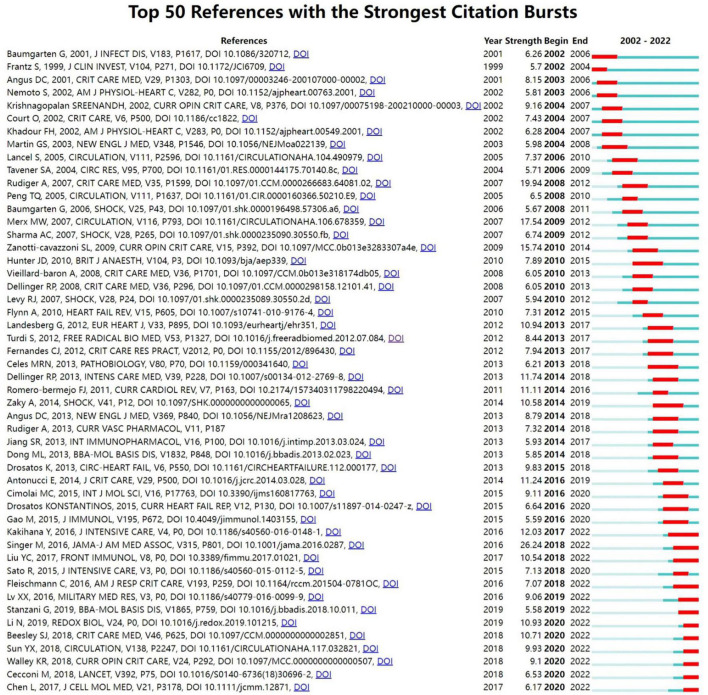
CiteSpace visualization map of top fifty references with the strongest citation bursts related to SIMD.

The term “reference burst” refers to a state in which the number of citations of this reference increases rapidly. In the visualization map of references with citation bursts performed by CiteSpace (Figure 7), the blue bars represent the timelines of corresponding articles from their publication to the present, and the red bars represent the timelines of citation bursts from their beginning to ending. The top fifty references with the strongest citation bursts started in 2002, and the reference with the strongest burst was from Singer M, published in 2016, and the burst strength of which was 26.24 as shown. Lastly and most importantly, the burst of 29 references (58%) in the top fifty references occurred in the last 10 years, and 12 references (24%) were still in the citation burst state in 2022, which implied that SIMD might attract sustained attention and have explosive development in the future.

### 3.6. Keywords co-occurrence and hotspots analysis

Key words are the core words used to express opinions in a paper and are also the prompt words for readers to consider whether it is necessary to read the paper. It means that research hotspots and directions in specific fields can be discovered and revealed through the co-occurrence of related keywords. A total of 820 different keywords were extracted from the 1,076 articles by CiteSpace, and the top 20 keywords appeared more than 100 times (Table 5). In research related to SIMD, the most common keywords were septic shock (375) and sepsis (372), followed by dysfunction (297), activation (241), expression (235), and etc. These topics are the hotspots of SIMD-related researches.

**TABLE 5:** The top 20 keywords related to SIMD.

Rank	Keywords	Count	Centrality	Rank	Keywords	Count	Centrality
1	septic shock	375	0.08	11	injury	153	0.06
2	sepsis	372	0.04	12	oxidative stress	152	0.04
3	dysfunction	297	0.08	13	mechanism	145	0.08
4	activation	241	0.08	14	inflammation	138	0.05
5	expression	235	0.05	15	nitric oxide	129	0.06
6	heart	206	0.09	16	heart failure	120	0.06
7	myocardial dysfunction	204	0.11	17	nf kappa b	118	0.07
8	cardiac dysfunction	203	0.08	18	severe sepsis	117	0.08
9	apoptosis	177	0.06	19	mortality	115	0.08
10	inhibition	160	0.1	20	necrosis factor alpha	102	0.07

**FIGURE 8:**
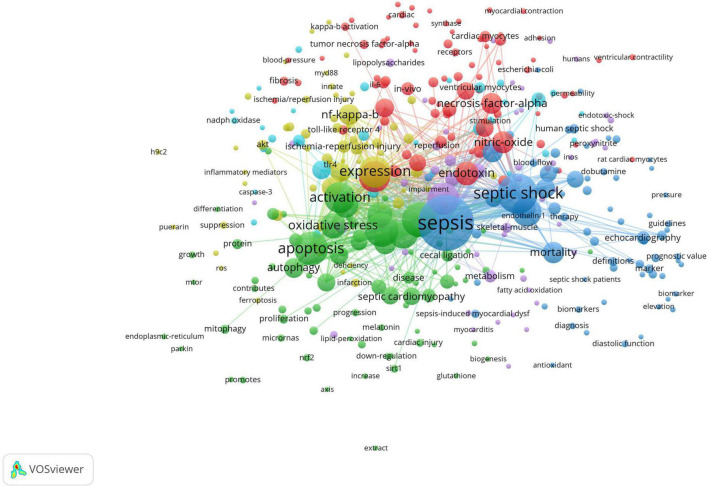
VOSviewer visualization map of keywords clustering analysis related to SIMD.

Clustering keywords on the node graph is helpful to explore the potential relationship between keywords, which was implemented *via* VOSviewer (minimum number of occurrences of a keywords ≥ 5) in this study. In Figure 8, each node represents a keyword, and different colors represent different clusters. All the keywords were grouped into six clusters, namely, six research directions or areas, which were cluster 1 (red), cluster 2 (green), cluster 3 (blue), cluster 4 (yellow), cluster 5 (purple), and cluster 6 (cyan). The main members of the red cluster were lipopolysaccharide, endotoxin, nitric-oxide, necrosis-factor-alpha, and cytokines. Them of the green cluster were dysfunction, apoptosis, inflammation, activation, and cardiac dysfunction. Them of the blue cluster were sepsis, septic shock, myocardial dysfunction, mortality and severe sepsis. While the keywords of the yellow cluster mainly included expression, nf-kappa-b, lps, mice and survival, those of the purple cluster included heart, receptor, metabolism, stress and *in vitro*. Lastly, the cyan cluster included cardiomyocytes, contractility, phosphorylation, kappa-b and calcium.

**FIGURE 9:**
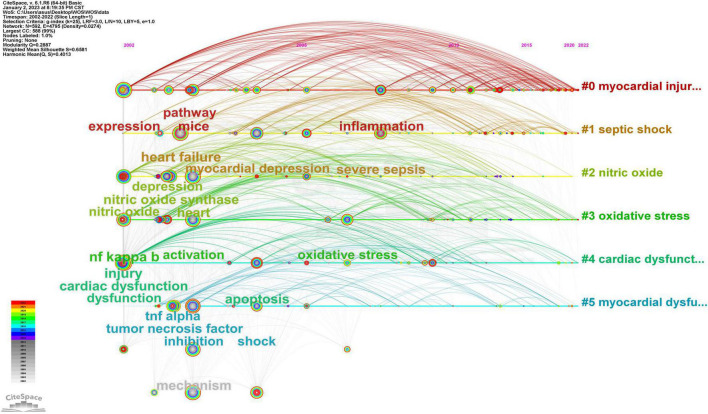
CiteSpace visualization map of timeline viewer related to SIMD.

A timeline viewer combines keyword clustering with time, which helps to show the evolution process of keywords in each cluster and the development trajectory of research hotspots in the whole field. CiteSpace was used to build the timeline viewer for these keywords (Figure 9). In this figure, the nodes on the timeline represent keywords at the time when they first appeared. The line between two points indicates that two keywords appear in the same article, while the color of the line indicates the year that the article was published. Therefore, it is obviously that most of the important keywords were proposed between 2002 and 2004. From 2002 to 2012, the research mainly focused on cardiac dysfunction in sepsis and its mechanistic relationship with inflammation. The key words of this period were mainly sepsis, septic shock, dysfunction, heart failure, myocardial dysfunction, cardiac dysfunction, injury, heart, expression, lipopolysaccharide, oxidative stress, inflammatory response, and etc. From 2013 to 2022, more studies focused on the relationship between SIMD and oxidative stress, as well as regulated cell death as a derivative. Some new keywords appeared in this period and became cores with parts of old ones, including autophagy, protect, myocardial injury, cardiomyocyte, cardiomyopathy, heme oxygenase 1, and pyroptosis.

## 4. Discussion

### 4.1. General information

The annual distribution of the articles helps to reflect the development trend of the SIMD research field (17). As shown in Figure 2, the number of articles published overall has increased. Specifically, in 2002, only 13 articles related to SIMD were published. From 2003 to 2015, SIMD was still in its infancy when the annual research results were limited, and the research heat remained at a low level. Since 2016, the research results of SIMD have begun to increase. The reason for this phenomenon might be that a new definition of sepsis had been proposed, which was called sepsis-3 and was considered as a more reasonable, accurate and applicable diagnostic criteria in the clinic (1, 18). Since 2018, the SIMD field has developed rapidly compared to before. The most successful case was *Beclin-1-Dependent Autophagy Protects the Heart during Sepsis*, published in 2018, demonstrating autophagy as a regulatory mechanism of SIMD, and this attracted more researchers to explore the mechanism of SIMD from the perspective of molecular biology rather than pathophysiology (19).

The field was very distinctive in its distribution of countries/regions and institutions. As shown in Table 1, the largest number of published papers was from China (551, 51.21%), followed by the USA (253, 23.51%), and the combined number of articles from the two countries accounted for 74.72% of the total (Figure 3). This indicates that China and the USA are the leading countries on SIMD. However, it was not consistent with centrality, a metric that measures the importance of each node in a network. The USA (0.38) had the best centrality and was in the first tier with Germany (0.37) and France (0.3), while China (0.17), Italy (0.15), and England (0.1) had lower ones. It seems that China should strengthen cooperation with other countries in this field to increase its influence (20). The low centrality might also be due to the fact that research results are distributed too widely across institutions. The top 10 institutions with the most published articles were all from China, but Wuhan Univ, which ranked the 1st, only published 37 articles (3.44%) with a centrality of 0.02. Meanwhile, China Med Univ (25, 2.32%) and Nanjing Med Univ (23, 2.14%) reached the highest centrality of 0.12. This information was consistent with the node diagram in Figure 4, showing that most institutions had no strong and tight cooperative relationships with others.

In the analysis of authors, Li Chuanfu, from East Tennessee State University, made the most significant contribution with 12 published articles, followed by Deng Wei (8, 0.74%) and Tang Qizhu (7, 0.65%) from Renmin Hospital and Cardiovascular Research Institute of Wuhan University and others (Table 2). LIU LI from the First Affiliated Hospital of Nanjing Medical University, who belonged to the same team as Li Chuanfu (Figure 5), was also in the top 10 with six published articles. It was noteworthy that Li Chuanfu and his team had been devoted to exploring the molecular mechanism and potential therapeutic targets of SIMD in recent years. Their main research directions, particularly in the last 10 years, have been phosphatidylinositol 3-kinase (PI3K)/protein kinase B (Akt) signaling pathway (21–23), apoptotic signaling (24, 25), and endothelial exosome (26, 27). They have proven in a mouse model of sepsis induced by cecal ligation and puncture that the PI3K/Akt is suppressed in SIMD (21), and the activation of this pathway by α-lipoic acid and toll-like receptor 9 helps prevents and treat SIMD (22, 23). They also suggests the regulatory role of nuclear factor κB and p53-mediated apoptosis on SIMD, as lentivirus expressing miR-125b/tumor necrosis factor receptor-associated factor 6 and glycolytic metabolism were believed to have a certain effect on SIMD by regulating such apoptotic signaling (24, 25). More importantly, the latest research results from Li showed that endothelial exosomes had a regulatory effect on SIMD, which was attributed to a macrophage pro-inflammatory response regulated by heat shock protein A12B, a protein predominantly expressed in endothelial cells, *via* suppression of adhesion molecule expression (26, 27). In conclusion, Li’s studies provided some ways for the prevention and treatment of SIMD. Besides, in the analysis of co-cited authors, only seven of the 1,058 cited authors were cited more than 150 times. The most frequently co-cited author was Rudiger Alain (238), as his work has provided the most influential research foundation in the field.

According to Table 3, the journals with the most articles about SIMD were *Shock* (68, 6.32%), followed by *Critical Care Medicine* (44, 4.09%), *American Journal of Physiology-Heart and Circulatory Physiology* (32, 2.97%), and etc. This indicated that the research hotspots of SIMD might mainly focus on clinical medicine and physiology in the last 20 years. As for co-cited journals, *Critical Care Medicine* (719) had the most citations and contributions, followed by *Circulation* (620), *Shock* (573), *American Journal of Physiology-Heart and Circulatory Physiology* (553), *Journal of Molecular and Cellular Cardiology* (502), and etc. Six of the top 10 co-cited journals belonged to Q1 in 2021, implying that high-impact journals provided credible theoretic or data bases for SIMD-related research (28). In addition, the dual-map overlay of journals on SIMD (Figure 6) showed that papers published in Molecular/Biology/Genetics journals were often cited by Molecular/Biology/Immunology and Medicine/Medical/Clinical journals, and that papers published in Health/Nursing/Medicine were also often cited by Molecular/Biology/Immunology. It seemed that both basic research and translational medicine were of great importance and attention to the SIMD research field currently.

### 4.2. Knowledge base

Knowledge base is composed of References with more co-citations, that are generally considered to provide a more widely recognized research basis for the corresponding field (20), these references can be obtained through co-cited analysis of references from literature (11). This study shows the top 10 references with the most co-citations (Table 4), a description of which is given in detail below.

Nineteen researchers from eighteen institutions, led by Mervyn Singer, published the most frequently cited reference in *JAMA* in 2016, titled *The Third International Consensus Definitions for Sepsis and Septic Shock (Sepsis-3)* (1). This study was a clinical guideline and expert consensus which revised the definition of sepsis to life-threatening organ dysfunction caused by a dysregulated host response to infection, called sepsis-3. Furthermore, this study revised the diagnostic criteria for sepsis based on the Sequential Organ Failure Assessment (SOFA) score, redecided the definition and diagnostic criteria for septic shock, and proposed a new sepsis screening standard called quick SOFA (qSOFA). These contributions helped to provide early diagnosis, management and treatment for sepsis patients or those at risk of sepsis, improve the consistency of relevant epidemiological studies and clinical trials, and attract researchers from non-infectious and immune fields to pay attention to the basic research of sepsis.

The second most cited reference was a review titled *Sepsis-Induced Myocardial Dysfunction: Pathophysiology and Management*, published by Yasuyuki Kakihana and others in 2016 in *Journal of Intensive Care* (29). This review outlined some characteristics of SIMD and assessed the most common underlying mechanisms, including global ischemia, myocardial depressants, cytokines and nitric oxide, damage-associated molecular patterns represented by histones and HMGB1.

The third reference was also a review, which was published by Yancun Liu in *Frontiers in Immunology* in 2017 and titled *Sepsis-Induced Cardiomyopathy: Mechanisms and Treatments* (30). This review summarized the mechanisms and treatments of *SIC*, especially from the perspective of molecular biology, such as toll-like receptor 4, nuclear factor κB, peroxisome proliferator-activated receptor, and etc. It is worth emphasizing that this review used another concept, sepsis-induced cardiomyopathy, which was first proposed by Elio Antonucci in 2014 in an attempt to define sepsis-induced myocardial depression as a disease rather than a pathological condition (31). However, this concept is still not widely recognized and used in clinical practice.

The fourth reference presented in Table 4 was published by Alain Rudiger in 2007 in *Crit Care Medicine* and had the highest centrality (0.36) (32). This review suggested that myocardial depression with reduced ejection fraction occurred in half of patients with sepsis, but that this reduction in cardiac function was reversible, so cardiomyocyte death was likely to be rare in such a process. The fifth one was published by Sarah J Beesley in 2018 and also in *Crit Care Medicine* (33). This review focused more on clinical applications and believed that treatment of sepsis-induced cardiac dysfunction had a more significant impact on prognosis, especially when the cardiac function of patients with sepsis decreased a lot. The sixth one was published by Yuxiao Sun in 2018 in *Circulation* (19). In this study, mouse models of lipopolysaccharide induced sepsis were used to conduct experiments which suggested that beclin-1-dependent autophagy might protect the heart from sepsis. Its discussion in conjunction with the fifth reference suggested that although cell death was rare in septic hearts, in all probability, regulating cell death might still have potential therapeutic effects on SIMD. The seventh was published by M W Merx in 2007 and also in *Circulation* (6), which did nearly the same as the second reference, but seemed to pay more attention to the role of regional myocardial ischemia or infarction secondary to coronary artery disease in SIMD. The eighth was published by Sergio L Zanotti-Cavazzoni in 2009 in *Current Opinion in Critical Care* (34), which also outlined some characteristics of SIMD and assessed the most common underlying mechanisms, and focused more on the effects of nitric oxide expression, mitochondrial dysfunction and apoptosis in SIMD. The nineth was published by Ning Li in 2019 in *Redox Biology* (35). This article reported that stimulators of interferon genes might activate NOD-like receptor protein 3 through phosphorylating type-I interferon regulatory factor 3 in a lipopolysaccharide-induced mouse sepsis model. Meanwhile stimulators of interferon gene deficiency could facilitate the alleviation of SIMD in mice. Besides, the NOD-like receptor protein 3 inflammasome was a classic agonist and biomarker of pyroptosis (36), which strongly suggested that pyroptosis was involved in the occurrence of SIMD. The tenth was published by Lukas Martin in 2019 in *Chest* (37). This review provided an overview of the most current, compared to the other top 10 references, understanding of SIMD in mechanisms, diagnosis, treatment and long-term outcome, and had a particular focus on pathophysiology.

Overall, the top 10 most co-cited references included one clinical guideline and expert consensus, seven domain reviews, and two original experimental articles, and nine of them were from Q1 journals with the highest IF (157.335) from *The Third International Consensus Definitions for Sepsis and Septic Shock (Sepsis-3)*. In these studies, the focus of the reviews was to summarize the known mechanisms involved in or regulating SIMD and potential therapeutic targets of SIMD, while the common point of the original articles was to demonstrate that the regulated cell death was related to the mechanism or regulation of SIMD.

### 4.3. The analysis of hotspots and frontiers

In bibliometrics, keywords are the most important research objects, because the hot spots and tendencies of the research field can be reflected by keyword co-occurrence analysis, knowledge structure and secondary fields can be obtained by improving keyword cluster analysis, and the evolution process and trend of hot spots can be gained by constructing a timeline view (38). As shown in Table 5, the top 20 keywords all appeared more than 100 times. They represented research hotspots in the SIMD field, of which the more representative ones were septic shock (375), sepsis (372), dysfunction (297), activation (241), and expression (235). The node size of the visualization map of keywords clustering analysis can also visually reflect high-frequency keywords in the field (Figure 8). However, the most important feature of keyword clustering analysis is to reveal research directions and knowledge structure (39). In this study, all the keywords were grouped into six clusters (Figure 8). Cluster 1 (red) was involved in inflammation in SIMD, cluster 2 (green) was related to sepsis-induced cardiac function change and its underlying mechanisms, and cluster 3 (blue) was with regard to epidemiological studies related to SIMD. Meanwhile, keywords of cluster 4 (yellow) were relative to animal models, mainly rats in the early period and mice in recent 10 years (40–44), of SIMD and *in vivo* experiments; of cluster 5 (purple) were in terms of cell models of SIMD and *in vitro* experiments; and of cluster 6 (cyan) were associated with molecular mechanisms of SIMD. In addition, the knowledge structure of SIMD could be considered as constructed from septic shock, sepsis, dysfunction, activation, expression, heart, myocardial dysfunction, cardiac dysfunction, and etc., which all belonged to cluster 1, cluster 2, and cluster 3. This indicated that, on the whole, research on SIMD in the last 20 years had focused more on clinical manifestations and epidemiology than on pathophysiology or molecular biology, which was probably due to the limitations of the previous definition of sepsis, Sepsis-2. From 2001 to 2016, Sepsis-2 was a widely accepted definition of sepsis, which defined sepsis to be the clinical syndrome defined by the presence of both infection and a systemic inflammatory response (45). This definition, derived entirely from clinical medicine, limited the depth of research on mechanisms of SIMD. Until the definition of sepsis was updated to Sepsis-3 in 2016 (1), the diagnosis and targeted treatment of patients with sepsis were more reasonable (46), while more researchers paid attention to pathophysiological process and related molecular mechanisms, such as endoplasmic reticulum stress (47), cardiovascular hyperpermeability (48), regulated cell death, macrophage polarization (49), mitochondrial dysfunction (50), fatty acid oxidation (51), calcium metabolism disorder (52), autonomic nervous dysregulation (53), and etc. Such a transformation was generally consistent with the results of the timeline viewer, but non-immune-related keywords began to appear in 2013, slightly earlier than the year of Sepsis-3 publication (Figure 9).

Citation bursts analysis is another way to detect emerging concepts and potential hotspots in a given research field in bibliometrics, applied to explore the abrupt change points and active research frontiers in the development of a field (54). Citation bursts analysis of references as circumstantial evidence was used in this study. The top fifty references with the strongest citation bursts were selected and shown in Figure 7. Currently, 12 of the top fifty references are experiencing a citation burst, while 6 literatures have been in this status for years (years: 2020–2022). Therefore, these 12 references were considered to reflect the latest research direction of SIMD. The orders of them based on the strength of the citation bursts (from high to low) were as followed. The references with the first (26.24) (1), second (12.03) (29), third (10.93) (35), fourth (10.71) (33), fifth (10.54) (30), and sixth (9.93) (19) highest strengths were coincident with the most frequently cited references described before in the section “Knowledge base.” This indirectly indicated that the research results related to SIMD had exploded in the last 3 years, which could corroborate each other with the annual growth trend (Figure 2). The seventh (9.1), published by Keith R Walley in 2018 in *Current Opinion in Critical Care* (55), and the eighth (9.06), published by Xiuxiu Lv in 2016 in *Military Medical Research* (56), were both reviews of SIMD focused on a physiologic and pathophysiological context. The nineth one (7.07) was a meta-analysis published by Carolin Fleischmann in 2016 in *American Journal of Respiratory and Critical Care Medicine*, which indicated that population-level epidemiologic data for sepsis was severely lacking in low- and middle-income countries. The tenth reference (6.53) was a review published by Maurizio Cecconi in 2018 in *Lancet* with the highest IF (202.731) of these references, which concluded that ongoing research aimed to improve the definition of patient populations to allow for individualized management strategies matched to a patient’s molecular and biochemical profile (57). The eleventh one (6.17) was an original article published by Lvyi Chen in 2017 in *Journal of Cellular and Molecular Medicine* (58). The results suggested that salidroside could be a potential therapeutic agent for the treatment of SIMD *via* regulating the PI3K/Akt pathway, which was exactly consistent with the results from Li Chuanfu and his team listed in the section “General information” (21). The last one (5.58) was another review published by Giacomo Stanzani in 2019 in *Biochimica et Biophysica Acta-Molecular Basis of Disease*, which focused on disrupted mitochondrial processes (59).

According to the above analyses, it is obviously that the research hotspots of SIMD initially focused on epidemiological features and the relationship with infection, and more recently focused on inflammatory response and molecular biological mechanisms. Recent studies have revealed some SIMD-related critical targets, including calpain (60, 61), heme oxygenase-1 (62, 63), Toll-like receptors (64, 65), NOD-like receptor protein 3 (66, 67), and silent information regulator 1 (68, 69). Oxidative stress and regulated cell death were likely to be the main directions and ideas for future studies on the molecular mechanisms of SIMD. However, although a few clinical studies have been conducted (70–72), evidence for SIMD-related clinical trials is still lacking.

### 4.4. Limitation

To begin with, the data were downloaded from the WOSCC database, meaning that literature not included in the database would not be included in the initial search, even if it was included in other WOS databases. Besides, the uneven quality of data might have a negative impact on the credibility of the map analysis. Meanwhile, the process of bibliometric analysis might lead to bias, as has been reported in other articles using the same method (73, 74). Nevertheless, visual analysis based on large volumes of literature lays an undoubted foundation for researchers to quickly understand the research hotspots, evolution processes and development trends in the SIMD field.

## 5. Conclusion

We collected the studies with high correlation with SIMD from 2002 to 2022 and analyzed the research hotspots, evolution process and development trend of SIMD. From the perspective of molecular biology, it is of outstanding value to study the pathogenesis and potential therapeutic targets of SIMD. According to the visual analysis by CiteSpace and VOSviewer, the number of papers concerning SIMD shows an increasing trend. The main countries concerned with this topic are China and the USA, while Wuhan University in China is the institution with the highest influence on achievements. However, cooperation and communication among countries and institutions need to be strengthened. Li Chuanfu has made significant contributions to the SIMD field. Most articles on SIMD have been published in internationally influential journals and focus on both basic research and translational medicine. Epidemiological features and the relationship with infection are key points for SIMD in the early period, but the molecular mechanisms of SIMD, particularly oxidative stress and regulated cell death, will be critical in the future.

## Data availability statement

The original contributions presented in this study are included in the article/supplementary material, further inquiries can be directed to the corresponding author.

## Author contributions

WZ and YY developed the topic and invited DL, HY, HZ, SL, YH, ZY, ZZ, and ZX. HY organized and edited the manuscript. ZZ and SL screened data and prepared figures and tables. DL and ZY prepared the section “General information.” ZX, YH, and HZ prepared the section “Knowledge base.” All authors have contributed to the content of this manuscript, reviewed and approved it for accuracy, completeness, and final manuscript.
